# Altered expression of microRNAs in the rat diaphragm in a model of ventilator-induced diaphragm dysfunction after controlled mechanical ventilation

**DOI:** 10.1186/s12864-021-07970-y

**Published:** 2021-09-18

**Authors:** Pengcheng Wang, Xianlong Zhou, Gang Li, Haoli Ma, Ruining Liu, Yan Zhao

**Affiliations:** 1 Emergency Center, Zhongnan Hospital of Wuhan University, 430071 Wuhan, China; 2 Hubei Clinical Research Center for Emergency and Resuscitation, Zhongnan Hospital of Wuhan University, 430071 Wuhan, China; 3 Department of Biological Repositories, Zhongnan Hospital of Wuhan University, 430071 Wuhan, China

**Keywords:** Controlled mechanical ventilation, Ventilator-induced diaphragm dysfunction, microRNA, Rat

## Abstract

**Background:**

Ventilator-induced diaphragm dysfunction (VIDD) is a common complication of life support by mechanical ventilation observed in critical patients in clinical practice and may predispose patients to severe complications such as ventilator-associated pneumonia or ventilator discontinuation failure. To date, the alterations in microRNA (miRNA) expression in the rat diaphragm in a VIDD model have not been elucidated. This study was designed to identify these alterations in expression.

**Results:**

Adult male Wistar rats received conventional controlled mechanical ventilation (CMV) or breathed spontaneously for 12 h. Then, their diaphragm tissues were collected for RNA extraction. The miRNA expression alterations in diaphragm tissue were investigated by high-throughput microRNA-sequencing (miRNA-seq). For targeted mRNA functional analysis, gene ontology (GO) analyses and Kyoto Encyclopedia of Genes and Genomes (KEGG) pathway analyses were subsequently conducted. qRT-PCR validation and luciferase reporter assays were performed. We successfully constructed a model of ventilator-induced diaphragm dysfunction and identified 38 significantly differentially expressed (DE) miRNAs, among which 22 miRNAs were upregulated and 16 were downregulated. GO analyses identified functional genes, and KEGG pathway analyses revealed the signaling pathways that were most highly correlated, which were the MAPK pathway, FoxO pathway and Autophagy–animal. Luciferase reporter assays showed that STAT3 was a direct target of both miR-92a-1-5p and miR-874-3p and that Trim63 was a direct target of miR-3571.

**Conclusions:**

The current research supplied novel perspectives on miRNAs in the diaphragm, which may not only be implicated in diaphragm dysfunction pathogenesis but could also be considered as therapeutic targets in diaphragm dysfunction.

**Supplementary Information:**

The online version contains supplementary material available at 10.1186/s12864-021-07970-y.

## Background

Many critically ill patients such as those with haemorrhagic shock [1], sepsis [2], or respiratory failure [3], including patients with COVID-19 and dysponea, are treated with ventilation, and ventilation may rapidly induce diaphragm disuse, weakness and atrophy [4]. This pathogenic process has been designated ventilator-induced diaphragm dysfunction (VIDD). Emerging evidence implies that it is the major complication of controlled mechanical ventilation (CMV). The development of VIDD is closely related to poor or adverse clinical prognoses in critically ill patients, who often suffer from an increased time of CMV, or even weaning failure.

The molecular and cellular mechanisms of VIDD have yet to be fully elucidated, and various studies of VIDD have linked it to autophagy [5], mitochondrial dysfunction [6], endoplasmic reticulum stress [7] and oxidative stress [8].

Recent research has focused on miRNAs, which are small endogenous RNA molecules with lengths of 19–23 nt. They bind to target sites (i.e., the 3’-UTR) in the noncoding regions of mRNA molecules to post transcriptionally regulate gene expression and protein synthesis. They are involved in the onset and pathology of many diseases. miRNAs have been reported to be involved in many diseases involving muscle atrophy. For instance, miR-21 was confirmed to be involved in skeletal muscle ageing and the decline in the potential for muscle regeneration [9]; miR-542-3p/5p was reported to affect patients with muscle atrophy via the acceleration of mitochondrial dysfunction [10]; and miR-182 was shown to be an important regulator of muscle atrophy-inducing genes under catabolic disease conditions [11]. It is clear that some miRNAs can regulate muscle atrophy in other pathogeneses; however, the actual effect of miRNAs in VIDD is not yet understood.

After CMV, accompanied by diaphragm dysfunction, the altered expression of diaphragm miRNAs might play a major role in modulating signaling pathways, gene expression and protein synthesis. All of the above factors might constitute a key mechanism of pathogenesis and be novel therapeutic targets.

The present study was aimed at idendifying differentially expressed miRNAs, finding novel therapeutic targets, and evaluating miRNA expression alterations in the rat diaphragm after CMV by high-throughput RNA sequencing. Various bioinformatic analyses were performed to predict miRNA functions according to Gene Ontologygene ontology (GO) analysis and Kyoto Encyclopedia of Gene and Genomes (KEGG) pathway analysis. A pathway relationship network was created based on the KEGG pathway analysis results and the identified DE (differential expressed) miRNAs to discover the relationships among them. Furthermore, nine miRNAs were randomly selected to perform quantitative reverse transcription-polymerase chain reaction (qRT-PCR) analysis, to validate the reliability of the RNA-seq results. Finally, a luciferase reporter assay was conducted to ascertain the links between miR-3571 and Trim63, miR-92a-1-5p and STAT3, and miR-874-3p and STAT3. This study revealed some novel and clear roles of these DE miRNAs in the diaphragm after CMV.

## Results

### Differential expression of miRNAs in diaphragm tissue after VIDD

**Fig. 1:**
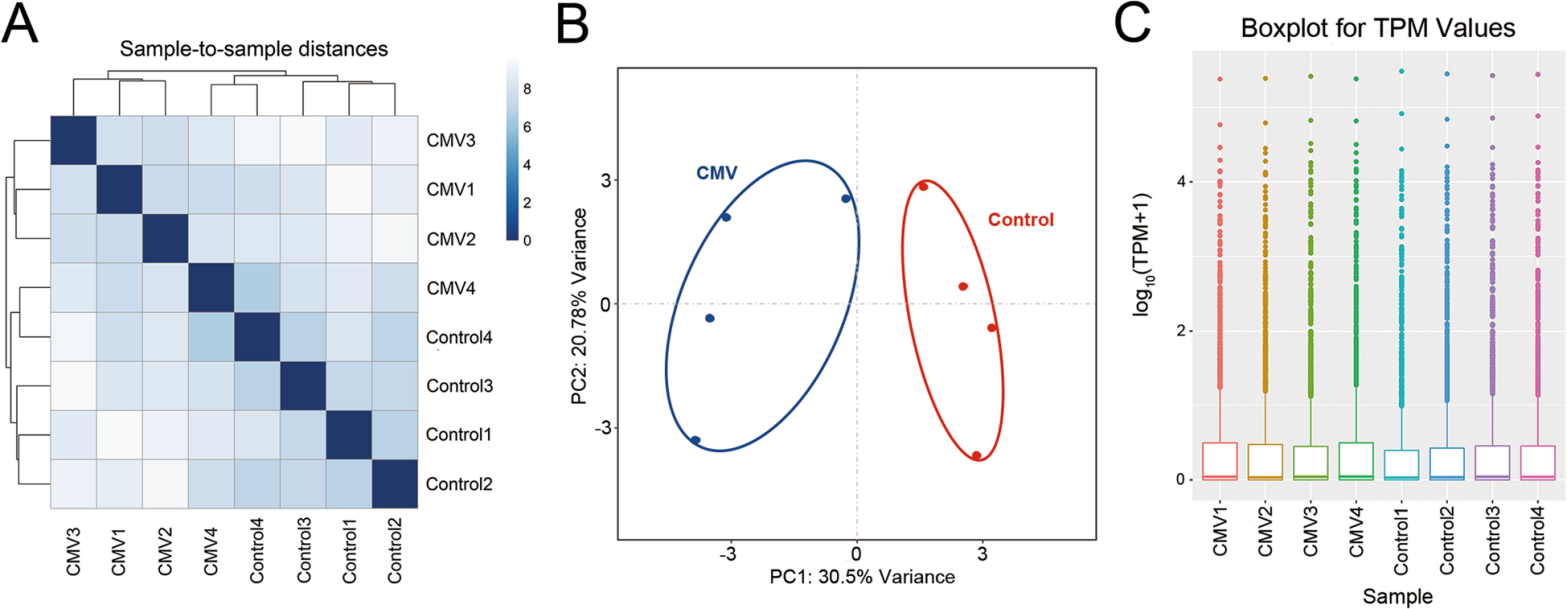
Correlations of samples. **A** The heatmap shows the sample-to-sample distance, and samples with high similarity were preferentially clustered together. **B** PCA analyses. **C** Boxplot of sample TPM values

**Fig. 2:**
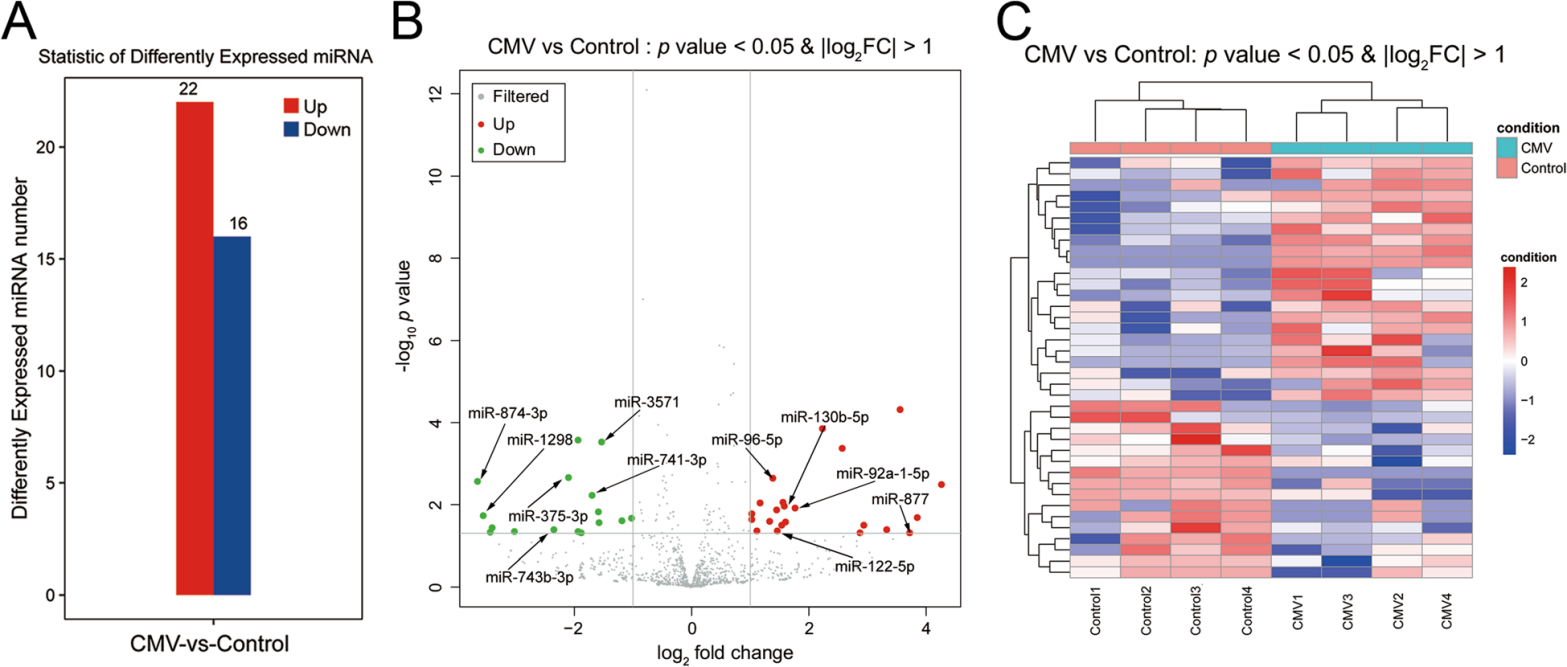
Differential expression of microribonucleic acids (miRNAs) in diaphragm tissue after mechanical ventilation. **A** Statistics of DE miRNAs. **B** Volcano plot of miRNA expression levels. The red dots indicate upregulated miRNAs with significantly differential expression, the green dots indicate downregulated miRNAs with significantly differential expression, and the grey dots indicate miRNAs without differential expression. The X-axis shows the log_2_ fold change values; the Y-axis shows the -log_10_ *p* values. **C** Heat map of DE miRNAs. Each row represents one probe set; each column represents one single sample. The dendrogram at the top reveals sample clustering; the dendrogram to the left reveals gene clustering

miRNAs were detected in four CMV and four control diaphragm tissue samples. The miRNA-seq reads of all samples are shown in Figure S1. The heatmap shows the distances between the samples. The samples with higher similarity were clustered together with high priority **(**Fig. 1**)**. We identified 38 significantly DE miRNAs according to the standard of a *p* value < 0.05 and |log_2_FC| > 1 in the expression level. Twenty-two of the 38 miRNAs were upregulated, and the other 16 were downregulated **(**Fig. 2A**)**. A volcano plot was created based on the mean miRNA expression values in each group, and the threshold was set at 2.0 for the fold change of expression **(**Fig. 2B**)**. A heatmap of 38 DE miRNAs was constructed to illustrate the discernible miRNA expression profiles **(**Fig. 2C**)**. The DE miRNAs were listed in Table 1. Detailed information on the differentially expressed (DE) miRNAs (|log_2_FC| > 1 and *p* < 0.05) were listed in Table S2.

**Table 1:** The differentially expressed miRNAs

miRNA ID	Log2FC	*p*-value	up/down	Sequence	Length
novel10_mature	2.585894365	0.000420713	Up	TCAGTGTGCTAGAGTCCTCGAAGA	24
novel10_star	3.578370892	4.88E-05	Up	TCCGAGGCTAGAGTCACGCTC	21
novel179_mature	2.886092162	0.048552039	Up	ATCTCGGTGGGACCGCCA	18
novel214_mature	1.572344804	0.008723536	Up	ACTGGACTTGGAGTCAGAAGA	21
novel231_mature	-3.001989158	0.04517486	Down	TTGGTACCTGTTTCCTGTTT	20
novel237_mature	2.953174649	0.031303233	Up	CGTGACTGTACCTGGTATT	19
novel30_mature	1.558144602	0.032272673	Up	ATATAGAGGATAATATAAATGT	22
novel316_mature	1.470591476	0.013690233	Up	AAAGTTTAACTTCTGCCA	18
novel31_mature	-1.566475963	0.027139728	Down	TATGGCGCTCCTCTGAGTAGA	21
novel322_mature	3.342200406	0.041283283	Up	TGACCCTCCTTTGCTCCTCAGG	22
novel379_mature	-3.384012887	0.037334425	Down	TCTCCGGCCTCTCGCGGGACCT	22
novel402_mature	1.13285745	0.043958532	Up	TCTGCTGACTGCCCATGGA	19
novel427_mature	2.252020331	0.000142047	Up	TCCGGCTGCGTCGGGCGTG	19
novel428_mature	3.867171168	0.020676937	Up	CCTGGGGCGGGCTGTGGGCTGTC	23
novel458_mature	-1.922705588	0.000263353	Down	CACTGGACTTGGAGTCAGAAGA	22
novel462_mature	4.274081175	0.003197987	Up	TTTGGTCTAAGGCTGGAACTTT	22
novel566_mature	1.045979075	0.022908305	Up	CATGGACGGTGTGAGGCCA	19
novel73_mature	-3.424160743	0.04782349	Down	TAAACCAGTCAGAGGATGGTAGG	23
novel75_mature	1.622119991	0.026499961	Up	ATGTAGTACTAAGTCTGTCACG	22
rno-miR-122-5p	1.47954028	0.04375855	Up	TGGAGTGTGACAATGGTGTTTG	22
rno-miR-1298	-3.537711659	0.018835052	Down	TTCATTCGGCTGTCCAGATGTA	22
rno-miR-130b-5p	1.596831274	0.010734419	Up	ACTCTTTCCCTGTTGCACTACT	22
rno-miR-147	1.347240696	0.025313554	Up	GTGTGCGGAAATGCTTCTGCTA	22
rno-miR-183-5p	1.047179965	0.016657263	Up	TATGGCACTGGTAGAATTCACT	22
rno-miR-200a-5p	-1.862942448	0.049359836	Down	CATCTTACCGGACAGTGCTGG	21
rno-miR-200c-3p	-1.572397551	0.014783046	Down	TAATACTGCCGGGTAATGATG	21
rno-miR-208b-3p	1.183001365	0.009169665	Up	ATAAGACGAACAAAAGGT	18
rno-miR-296-3p	-1.170361668	0.024842243	Down	GAGGGTTGGGTGGAGGCTCTCC	22
rno-miR-3571	-1.516247884	0.00029504	Down	TACACACTTCTTTACATTCCATA	23
rno-miR-375-3p	-2.086674249	0.002167474	Down	TTTGTTCGTTCGGCTCGCGTGA	22
rno-miR-377-5p	-1.005516248	0.021749086	Down	AGAGGTTGCCCTTGGTGAATTC	22
rno-miR-501-5p	-1.925263887	0.045437012	Down	AATCCTTTGTCCCTGGGTGA	20
rno-miR-741-3p	-1.685014571	0.005957175	Down	AAAGATGCCACGCTATGTAGAT	22
rno-miR-743b-3p	-2.333443622	0.040744086	Down	GAAAGACACCATACTGAATAGA	22
rno-miR-874-3p	-3.638678583	0.002704144	Down	CTGCCCTGGCCCGAGGGACCGA	22
rno-miR-877	3.735698614	0.048157151	Up	GTAGAGGAGATGGCGCAGGG	20
rno-miR-92a-1-5p	1.784733476	0.012196833	Up	AGGTTGGGATTTGTCGCAATGCT	23
rno-miR-96-5p	1.398245576	0.002265536	Up	TTTGGCACTAGCACATTTTTGCT	23

*FC* fold change

### GO analysis of DE miRNA-targeted genes

**Fig. 3:**
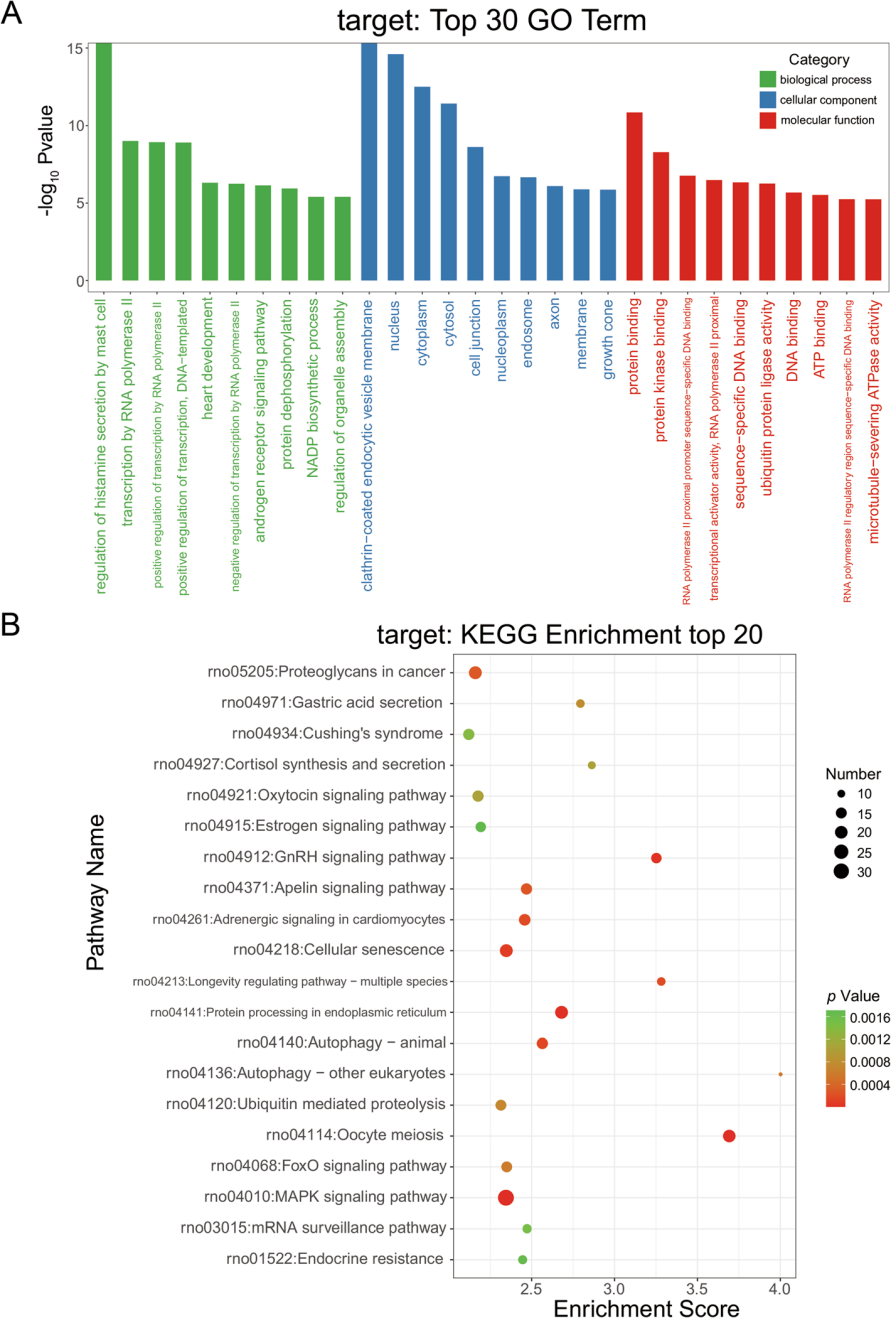
Gene Ontology (GO) analysis and Kyoto Encyclopedia of Genes and Genomes (KEGG) pathway analysis. **A** GO analysis of DE miRNAs with the top 30 genes according to the -log_10_ *p* value for each GO term. **B** KEGG pathway analysis of differentially expressed miRNAs, with the top 20 enrichment scores

The identified genes (mRNAs) were subjected to GO functional analysis to explain and hypothesize the functions of the DE miRNAs **(**Fig. 3A**)**. These GO terms applied a correction for multiple testing. The type of correction was Benjamini and Hochberg (BH) of False Discovery Rate (FDR). Detailed information on the differentially enriched GO analysis is listed in Table S3. The results of GO analysis can be classified into three major categories: biological process (BP), cellular component (CC), and molecular function (MF). These columns are presented in the figure in descending order of -log_10_ *p* values. The BP results showed that the DE miRNAs were strongly related to the regulation of histamine secretion by mast cells, transcription by RNA polymerase II, positive regulation of transcription by RNA polymerase II, and so on. Among the GO CC terms that were significantly enriched in DE miRNAs, the miRNAs were associated with the clathrin-coated endocytic vesicle membrane, nucleus, cytoplasm, cytosol, cell junction. In the MF category, the DE miRNAs were associated with terms such as protein binding, protein kinase binding. The distribution of DE miRNA target genes at GO level2 is shown in Figure S2A.

### KEGG pathway analysis of DE miRNA-targeted genes

The dot plot of the identified KEGG pathways shows the top 20 significantly enriched pathways in order of enrichment score values. These KEGG terms applied a correction for multiple testing. The type of correction was Benjamini and Hochberg (BH) of False Discovery Rate (FDR). Detailed information on the differentially enriched KEGG pathways is listed in Table S4. The pathways influenced by the variation in miRNAs and targeted mRNAs in diaphragm tissue after VIDD could be predicted. The top 20 pathways are showed in Fig. 3B, and included the Oocyte meiosis, MAPK signaling pathway, Protein processing in endoplasmic reticulum, GnRH signaling pathway, Cellular senescence, Autophagy–animal.

According to the KEGG classification, the numbers of proteins or genes identified at different functional levels, including cellular processes, environmental information processing, genetic information processing, human diseases, metabolism and organismal systems, were quantified. The signal transduction of environmental information processing category appeared to include the highest percentage of all identified genes, which may imply a major role in VIDD pathogenesis. The categories with the next highest percentages of the identified genes were infectious diseases and cancers of Human Diseases, Endocrine system of Organismal Systems. The relatively high percentages if genes in these categories suggested that they play important roles in this pathogenic process. (Figure S2B).

**Fig. 4:**
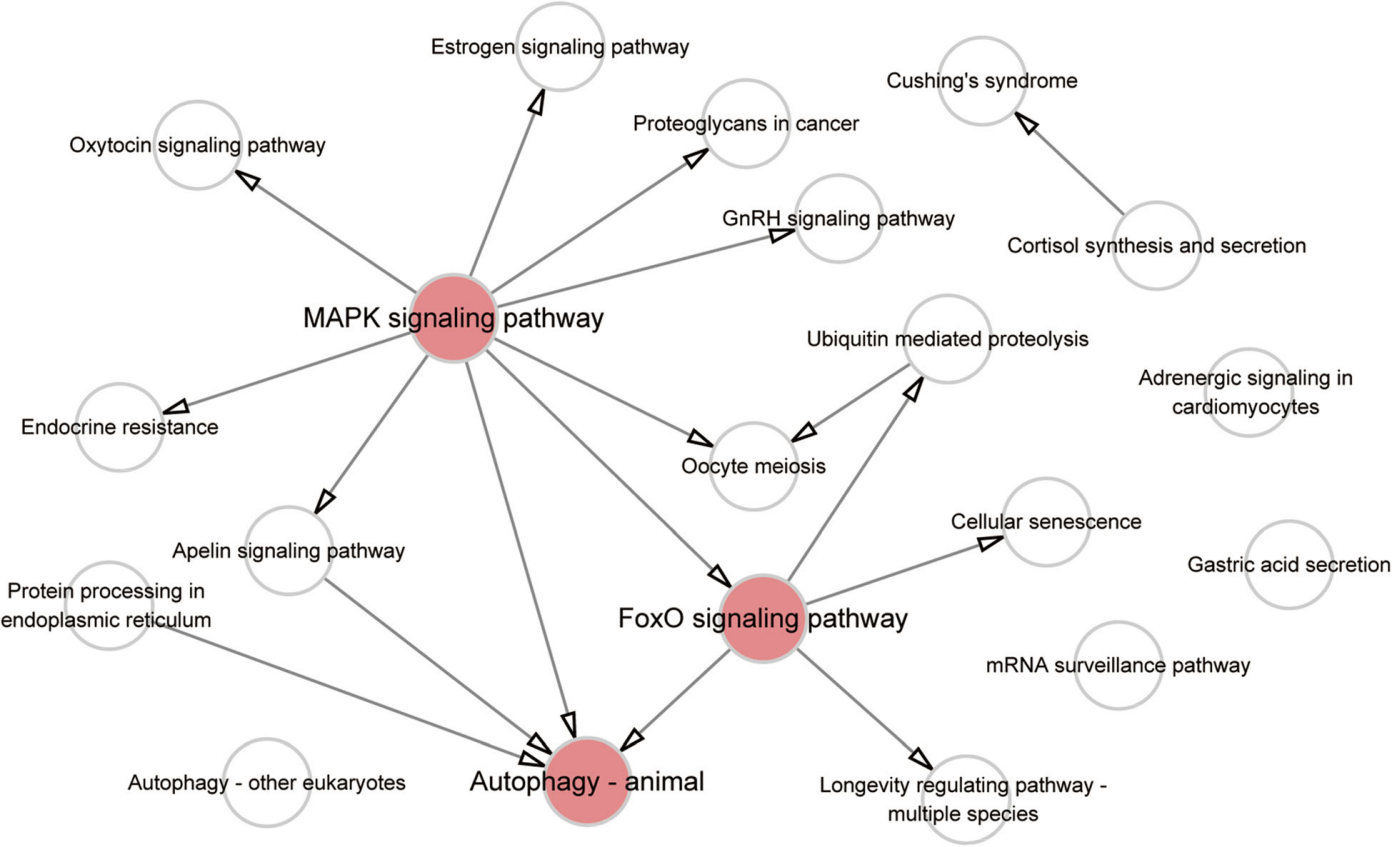
Pathway relationship network analysis of the top 20 pathways enriched with miRNAs. The network was constructed based on Kyoto Encyclopedia of Genes and Genomes (KEGG) pathway analysis and a KEGG database search

The pathway relationship network of the top 20 pathways enriched in DE miRNAs was drawn (Fig. 4). This network implied that among all the pathways, three particular pathways may have an upstream regulatory effect. These pathways were the MAPK signaling pathway, the FoxO signaling pathway and Autophagy–animal pathway.

### Validation of the reliability of miRNA-seq data by qRT-PCR

**Fig. 5:**
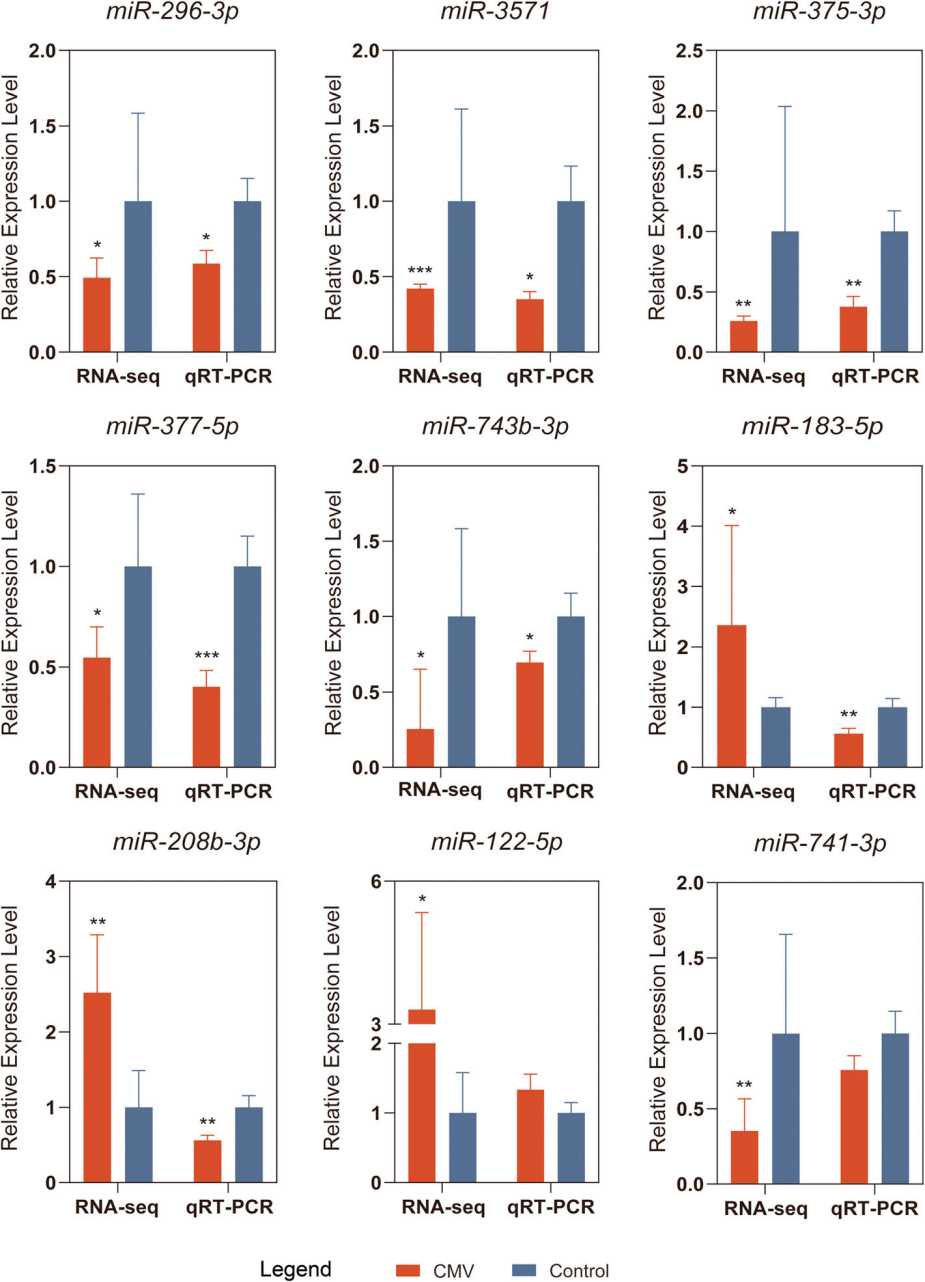
Quantitative reverse transcription polymerase chain reaction (qRT-PCR) validation results and RNA-seq results for the 9 selected microribonucleic acids. *, *p* < 0.05; **, *p* < 0.01; ***, *p* < 0.001

Nine DE miRNAs were randomly selected to verify the accuracy of miRNA-seq results. U6 was set as an internal control. Comparisons between the expression levels of these miRNAs determined by miRNA-seq or qRT-PCR are shown in Fig. 5. The expression levels revealed by qRT-PCR analysis were calculated by the 2^−ΔΔCT^ method. The data were expressed as mean ± SD values. The results were significant for seven of the nine miRNAs (*p* < 0.05). The variation trends of miR-296-3p, miR-3571, miR-375-3p, miR-377-5p, and miR-743b-3p were in accordance with the miRNA sequencing data, while those of the two other miRNA—miR-183-5p and miR-208b-3p—were not. Although the differences were nonsignificant (*p* value > 0.05), the results for two other miRNAs (miR-122-5p and miR-741-3p) implied concordance with the miRNA-seq results (data not shown). In total, the verifiable validation rate was 7/9. This validation result showed that the miRNA-seq results were reliable.

### Luciferase validation

**Fig. 6:**
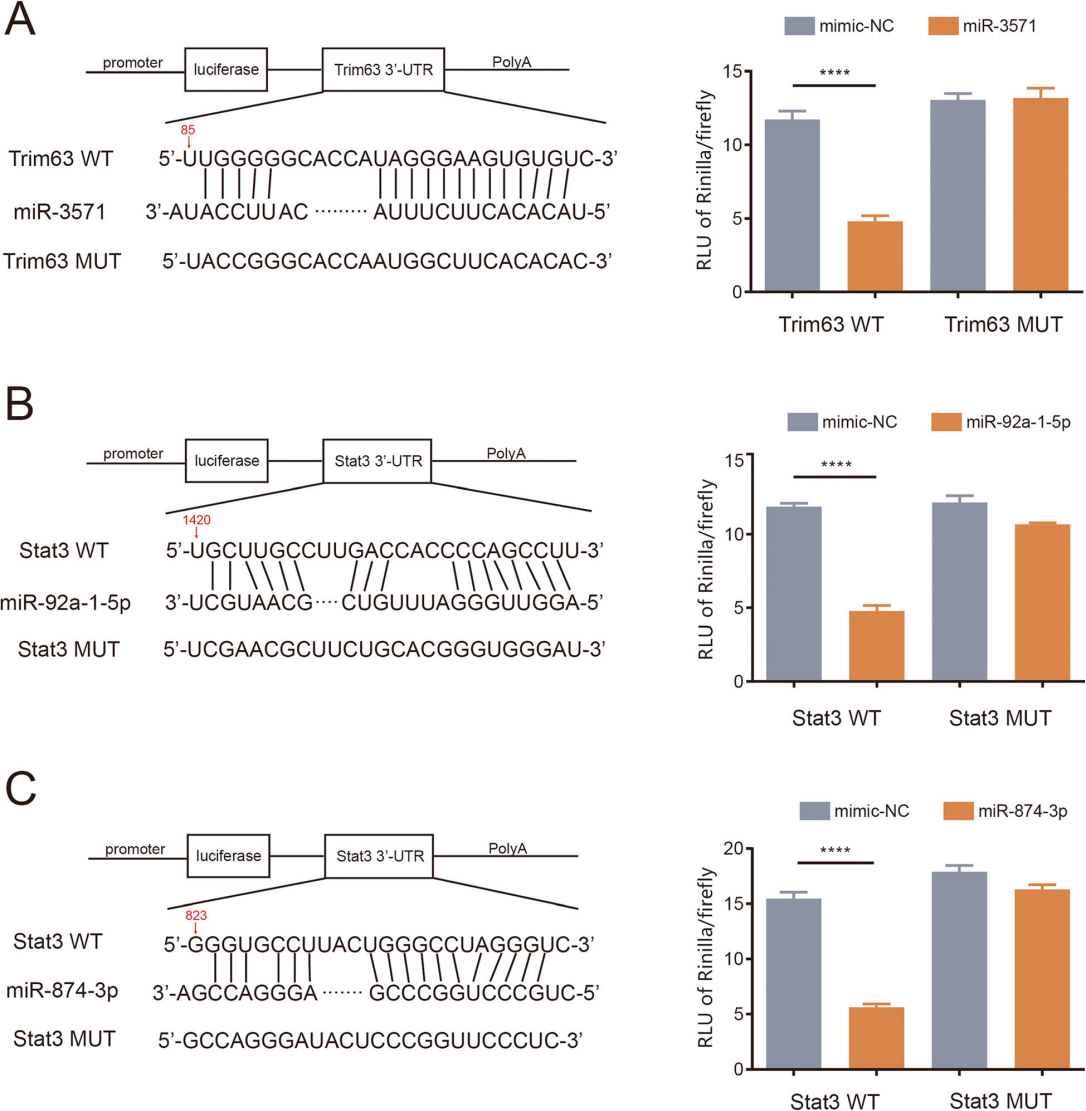
Luciferase reporter assay. **A** miR-3571 and Trim63, **B** miR-92a-1-5p and STAT3, **C** miR-874-3p and STAT3

MicroRNAs exert their inhibitory effects on mRNAs by binding to their 3’-UTRs. Multiple direct targets of miRNAs have been described in the literature to date. We searched for novel targets of miRNAs within mRNAs that could potentially account for the variation in the expression of miRNAs in diaphragm tissue after the rats were subjected to controlled mechanical ventilation. On the basis of bioinformatic analysis in the miRDB (http://mirdb.org), TargetScan (http://www.targetscan.org) and Diana (http://diana.imis.athena-innovation.gr/DianaTools) databases as well as previous studies, we selected three miRNA-mRNA interactions for further analysis and tested them by using luciferase reporter assays. We separately cloned the 3’-UTRs of Trim63 and STAT3 containing miR binding sites, downstream of the luciferase reporter gene. The normalized luciferase activity was significantly reduced upon cotransfection with miR-3571 and Trim63 WT and upon cotransfection with either miR-92a-1-5p or miR-874-3p and STAT3 WT. The cells that were cotransfected with MUT and either mimic-NC or mimic miRNA showed no significant differences in each group **(**Fig. 6**)**. These results indicate that STAT3 is a direct target of both miR-92a-1-5p and miR-874-3p and that Trim63 is a direct target of miR-3571.

## Discussion

In the present study, we revealed the altered expression profile of miRNAs in the rat diaphragm tissue after CMV for the first time by high-throughput miRNAome sequencing followed by GO and KEGG pathway analysis. We made several novel findings which may accelerate the understanding of post-CMV molecular and intermolecular alterations coupled with the critical signaling pathways in which these molecules participate.

First, we showed that miRNA expression changed after CMV, and these changes may reveal the physiological and pathophysiological processes occurring after CMV and may offer potential therapeutic targets for VIDD. We identified a total of 38 DE miRNAs in diaphragm tissue after CMV, among which 22 were upregulated and 16 were downregulated. The miRNAs and their Fold Change level were listed in Table 1. They might participate in the onset, progression, and development of VIDD. To our knowledge, only a few previous studies have illustrated the involvement of various miRNAs from different sources in VIDD. For example, Moroz, N., et al. [12] reported that some autophagy-related regulated miRNAs, such as miR-106b, miR-20a, miR-101a, miR-376, miR-204 and miR-93, show lower expression in diaphragm tissue during mechanical ventilation. In other diseases, the downregulation of miR-1, miR-133, miR-486 and miR-206 has been observed in the diaphragms and limb muscles of mice with lung cancer cachexia [13]. The miRNA expression pattern of their research was different from ours. We propose that this discrepancy may be due to sample and/or disease disparities, the miRNA expression alteration in induced diaphragm might be regulated by experimental conditions or species difference.

Several DE miRNAs that we identified have been reported in previous studies. For instance, miR-122-5p was up-regulated in our study, it has been reported to participate in regulating glioma [14, 15], hepatocellular carcinoma [16–20], myocardial injury [21], and osteoarthritis [22]. It has also been reported in studies of diseases of muscle dysfunction or degeneration, such as in normal skeletal muscle. The downregulation of miR-122-5p has been found in skeletal muscle fibrosis, and miR-122-5p overexpression has been shown to retard the fibrotic process by targeting the TGF-β/Smad signaling pathway [23]. Another study showed a similar result: miR-122-5p may restore myogenesis [24]. These combined results show the potential therapeutic effects of this miRNA in translational studies. Additionally, miR-296-3p was down-regulated in our study, it has been reported in several types of tumors, such as neurofibromatosis [25], lung adenocarcinoma [26], and prostate cancer [27]. As well as miR-375-3p and miR-874-3p, miR-375-3p was found to suppress tumorigenesis by targeting YAP1 and SP1 in colorectal cancer cells [28], and alleviate the severity of inflammation through targeting YAP1/LEKTI pathway in HaCaT cells [29], miR-874-3p was found as an anti-oncomir in esophageal squamous cell carcinoma via targeting STAT3 [30]. As to the down-regulated miR-3571, it was reported in associated with selenium deficiency-induced cardiac dysfunction [31]. These miRNAs have not been previously reported to be associated with diaphragm dysfunction to our knowledge. However, in these diseases, the target genes they regulate are also reported in diaphragm function, or the similarity of some disease with VIDD (cardiac dysfunction), suggesting that these miRNAs may also have therapeutic effect on VIDD. Thus, our sequencing results may offer some novel findings.

To acquire insight into the prospective functions of the DE miRNAs, we performed KEGG pathway analyses to reveal the roles of the differentially expressed miRNAs in several biological pathways. Among these pathways, the MAPK signaling pathway and FoxO signaling pathway were the only two pathways that were correlated with environmental information processing and signal transduction. Based on the results of KEGG library database search [32], we constructed a pathway relationship network. This network showed the connections of the 20 most enriched signaling pathways, from which we found that the MAPK signaling pathway seemed to be the most important upstream signaling pathway, and that the FoxO signaling pathway was an important downstream pathway of MAPK. The autophagy–animal pathway was also identified. Several studies have shown the roles and prospective therapeutic effects of activating the FoxO signaling pathway [33] and autophagy [11, 34] in VIDD and have determined the underlying mechanisms.

The MAPK signaling pathway has been fully elucidated for quite some time; it is composed of a group of serine/threonine protein kinases involved in cellular signal transduction that regulate many cellular functions and affect many human diseases [35]. Previous studies have proven the effects of MAPK signaling on tumor metastasis [36], osteoarthritis [37], traumatic brain injury [38], and certain inflammatory diseases [39]. For example, MAPK signaling is activated in the diaphragm tissue of mice with Duchenne muscular dystrophy [40]. In a model of nutritional deprivation-induced cachexia and muscle atrophy, the phosphorylation of MAPK/ERK was shown to be significantly downregulated in the diaphragm [41]. Another study also showed that MAPK/p38 and MK2 were associated with limb and diaphragm muscles [42]. Regarding other diseases, research on osteonecrosis showed that exosomes carrying overexpressed miR-122-5p regulated the MAPK signaling pathway in the femoral head [43]. Other studies showed that the forced expression of miR-122-5p induced MAPK signaling in breast cancer [44]; that miR-200a-5p regulated Se deficiency-induced myocardial necroptosis, which was hypothesized to be mediated by MAPK [45]; and that miR-200c-3p inhibited HMGB3, which can activate MAPK signaling, in glioblastoma [46]. This evidence collectively shows that several miRNAs identified in our study are related to the MAPK signaling pathway.

Based on previous studies [47], we selected several VIDD-related genes(e.g., Trim63 and STAT3). It is well established that Trim63 is upregulated in rats with VIDD after CMV [48], and JAK-STAT3 signaling is also altered [49]. On the basis of database searches, we speculated that they were regulated by several DE miRNAs found in our miRNA-seq results. In particular, STAT3 was regulated by miR-92a-1-5p and miR-874-3p, and Trim63 was regulated by miR-3571. Luciferase reporter assays were performed to verify the link between miRNAs and these genes. The results showed that miR-3571 can negatively regulate Trim63 and that miR-92a-1-5p and miR-874-3p can negatively regulate STAT3. Therefore, we hypothesized that these miRNAs may serve as critical targets in VIDD pathogenesis and that their upregulation may be a novel potential therapy for VIDD, giving rise to the opportunity for translation into human populations.

Finally, we predicted miRNA and targeted mRNA network according to the database and bioinformatic analysis criteria (Table S5) and constructed a miRNA-mRNA network (Figure S3) by Cytoscape (v3.6.0). Briefly, the results showed that miR-296-3p, miR-147, miR-200c-3p and miR-877 regulated relatively few mRNAs, whereas miR-3571, miR-743b-3p and miR-96-5p regulated more mRNAs. According to this analysis, the roles of these miRNAs and related mRNAs in VIDD may merit further research.

## Conclusions

In the present study, we identified a series of DE miRNAs in diaphragm tissue after CMV-induced diaphragm dysfunction for the first time; these miRNAs may be linked to physiological and pathological processes during VIDD. We also estimated the potential roles of signaling pathways and miRNA-mRNA interactions. This study showed that miRNAs in diaphragm tissue are altered after VIDD, which implies that they may merit consideration as therapeutic targets in VIDD.

## Methods

### Animals

SPF-level adult male Wistar rats were obtained from Charles River Laboratories (Beijing, China); their weight varied from 450 to 550 g. All animal studies were performed in the Bio-Safety Level III Laboratory of Wuhan University (Wuhan, Hubei, China). Animal experiments were approved by the Animal Experiment Center and the ethics committee of Zhongnan Hospital of Wuhan University and were conducted following the National Institutes of Health Guide for the Care and Use of Laboratory Animals. The rats were housed in dedicated cages under controlled conditions (temperature: 25 °C ± 2 °C; relative humidity: 50 % ± 5 %) with a 12:12 light-dark cycle. Water and food were provided *ad libitum*.

### Controlled mechanical ventilation (CMV) model and ventilator-induced diaphragm dysfunction (VIDD)

Eighteen animals were randomly divided into two groups by using a table of random numbers: (1) a control group (*n* = 9), in which animals underwent a sham operation and were not ventilated; and (2) the CMV group (*n* = 9), in which animals received CMV for 12 h. The animal model of mechanical ventilation was established in accordance with a previous study [50]. Briefly, animals were anaesthetized with sodium pentobarbital (40 mg/kg, ip.). After successful anaesthetization, rats were fixed on a recirculating heating blanket. They were tracheostomized and then connected to a volume-driven small animal ventilator (VentElite, USA). The ventilator parameters were set as follows: the respiratory rate (RR) was set at 55 to 60 rpm, and the tidal volume (TV) was set at 5 mL/kg body weight. The breathing air of the rats was humidified with sterilized saline and enriched with oxygen. Arterial blood gas analysis was conducted every 2 h during ventilation to maintain at a PaCO_2_ between 35 and 45 mmHg and a PaO_2_ between 80 and 100 mmHg during the entire 12 h ventilation period to avoid ventilator-induced systemic hypoxia. Blood pressure (BP) and heart rate (HR) measured at the tail artery by tail cuff plethysmography (BP-2010 Series Blood Pressure Metre, Softron, Japan) were recorded in real time. We selected and cannulated the right jugular veins of the rats for the continuous infusion of normal sterilized saline (Baxter, Deerfield, IL) (1 mL/kg/h) and pentobarbital sodium (10 mg/kg/h) with an electric pump. The body temperature of the rats was maintained at 37 °C during the study by external warming through a homeothermic blanket system [47]. The rats were conventionally euthanized by the intraperitoneal injection of an overdose of pentobarbital sodium (100 mg/kg) before the diaphragm was removed. Among all the samples, eight (four from the CMV group and four from the control group) were used for miRNA-seq, and ten (five from the CMV group and five from the control group) were selected for qRT-PCR validation.

### MicroRNA sequencing and subsequent bioinformatic analysis

Total RNA was extracted from diaphragm tissue samples using a miRNeasy Mini Kit (Qiagen, Cat. No. 217004). RNA integrity was assessed using an Agilent 2100 Bioanalyzer (Agilent Technologies, USA). After this assessment, the miRNA samples with an RNA integrity number (RIN) ≥ 7 were selected for subsequent analysis. TruSeq Small RNA Sample Prep Kit (Cat. No. RS-200-0012, Illumina, USA.) was used for library construction. Small RNA sequencing and analysis were carried out by OE Biotech Co., Ltd. (Shanghai, China). The basic reads were transformed into raw data by base calling, and their sequences were obtained on the Illumina HiSeq 4000 sequencer platform. Low-quality reads were filtered. Reads with 5’ primer contamination and poly (A) tails and those without 3’ adapters were removed. In addition, reads without insert tags and those that were shorter than 15 nt or longer than 41 nt in the raw data were filtered to obtain the clean reads. The minimal read count was set as 1.

First, the clean reads were used for length distribution analysis in the reference genome. These clean reads were aligned, subjected to BLAST database analysis [51], and subjected to searches against the Rfam v.10.1 (http://www.sanger.ac.uk/software/Rfam) [52, 53] and GenBank databases (http://www.ncbi.nlm.nih.gov/genbank/). The noncoding RNAs were annotated as tRNAs, rRNAs, small nuclear RNAs (snRNAs), and small nucleolar RNAs (snoRNAs), and were excluded from the analysis. Known miRNAs were identified by alignment against the miRBase v.22.1 database (http://www.mirbase.org/) with Bowtie (v1.1.1) [53], and their expression levels were normalized by the transcript per million (TPM) method, according to the formula N/M*10^6^, where N presents the reads for each miRNA, and M presents the reads in each sample. Unannotated small RNAs were analysed with miRDeep2 [54] to predict the structure of novel miRNAs, based on the pre-hairpin structure and the criteria of the miRbase database.

DE miRNAs were identified as those meeting the threshold of a *p* value < 0.05 and a fold change > 2.0. The fold change and *p* values were calculated with the DEG algorithm (R, DESeq package) [55]. The target mRNAs of DE miRNAs were predicted by using the following databases: miRDB (http://mirdb.org), TargetScan (http://www.targetscan.org) and Diana (http://diana.imis.athena-innovation.gr/DianaTools), and an intersection of all the target genes listed in the databases was selected.

GO (http://www.geneontology.org) enrichment and KEGG (http://www.genome.jp/kegg) pathway enrichment analyses of the DE miRNA-targeted genes were calculated based on the hypergeometric distribution (R, stats package) [56].

### qRT-PCR analysis

We performed qRT-PCR to prove the accuracy of the miRNA-seq data. RNA samples were reverse transcribed into cDNA using a Mir-X miRNA First Strand Synthesis Kit (638313, TaKaRa, Osaka, Japan). Briefly, RNA samples were diluted in RNAse-free DEPC water and mixed with mRQ buffer and mRQ enzyme. Then they were incubated for 1 h at 37 °C, followed by 85 °C for 5 min to inactivate the enzymes on a thermal cycler. After reaction termination, the cDNA samples were diluted with a 9-fold volume of DEPC water. Real-time PCR was conducted using TB Green Premix Ex Taq II (Tli RNaseH Plus) (RR820A, TaKaRa, Japan). First, cDNA samples, primers, and DEPC water were mixed in 96-well plates, and qRT-PCR was performed on a CFX Connect Real-Time PCR System (Bio-Rad; CFX Maestro 1.0 software). The thermal cycling program was set as follows: 95 °C, 30 s; 45 PCR cycles (95 °C, 5 s; 60 °C, 30 s [plate read]); and 95 °C, 10 s, with melt curve analysis at the end of the program. The sequences of the primers used in this experiment are shown in Table S1.

### Luciferase reporter assay

We selected the trim63 and STAT3 genes, which have been widely reported in VIDD studies, and the identified DE miRNAs for which not reported in VIDD yet. The miRNA-mRNA connections were selected according to the TargetScan database (http://www.targetscan.org) and previous studies. The links between miR-3571 and Trim63, miR-92a-1-5p and STAT3, and miR-874-3p and STAT3 were determined, and a luciferase reporter assay was conducted for verification. Briefly, the 3’-UTRs of Trim63 and STAT3 with putative miRNA binding sequences (Rat Genome Database, https://rgd.mcw.edu) [WT (wild type) or MUT (mutant type)] were cloned, and the base sequences were confirmed by sequencing. The plasmid sequences carried in the vector were consistent with the expected sequences, confirming the successful construction of the vectors. The psiCHECK-2 vector (Uptbio, China) was double digested, and the target fragment was linked to the linearized vector at the insertion sites of XhoI and NotI. Lipofectamine 3000 (Thermo Fisher Scientific) was used to cotransfect the resulting constructs (0.8 µg) with mimic-NC, miR-3571, miR-874-3p, or miR-92a-1-5p (each 100 nM) into HEK293 reporter cells (Procell, Wuhan, China) plated in 24-well plates. Cells were incubated at 37 °C for 24 h, and the relative light unit (RLU) values of firefly luciferase and Renilla luciferase were subsequently detected and recorded with a Luminometer (Lux-T020, BLT). The ratio of Renilla luciferase to firefly luciferase in each group was calculated.

### Statistical analysis

The statistical analyses were performed in SPSS 24.0. Data are expressed as the mean ± SD format. Student’s *t*-test was selected to evaluate differences between two groups in qRT-PCR. A *p *value < 0.05 was considered to indicate a significant difference.

## Supplementary Information

**Additional file 1: Table S1. **Primers sequences used in this study.

**Additional file 2: Table S2. **Detailed information for differentially expressed (DE) miRNAs (|log_2_FC| > 1 and *p* < 0.05).

**Additional file 3: Table S3.** Detailed information for the differentially enriched GO term analysis.

**Additional file 4: Table S4. **Detailed information for the differentially enriched KEGG pathways.

**Additional file 5: Table S5. **Interaction of miRNAs and mRNAs.

**Additional file 6: Figure S1. **Novel miRNA categories.

**Additional file 7: Figure S2. **(A) GO level2 distribution of different miRNAs. (B) KEGG level2 distribution of different miRNAs.

**Additional file 8: Figure S3. **mi-mRNA relation network analysis. The blue circles indicate targeted mRNAs, and the red circles indicate the DE miRNAs related with these mRNAs. The size of the circles represents the number of connections.

## Data Availability

All of the raw data have been uploaded to the Sequence Read Archive (SRA),and the accession number is SRX11758233-11758240. The data can be accessed at https://www.ncbi.nlm.nih.gov/bioproject/PRJNA753226. The miRNA database we refer to can be found at http://www.mirbase.org/cgi-bin/browse.pl?org=rno, the Genome Database can be found at ftp://ftp.ncbi.nlm.nih.gov/genomes/all/GCF/000/001/895/GCF_000001895.5_Rnor_6.0/GCF_000001895.5_Rnor_6.0_genomic.fna.gz, and the mRNA Database can be found at ftp://ftp.ncbi.nlm.nih.gov/genomes/all/GCF/000/001/895/GCF_000001895.5_Rnor_6.0/GCF_000001895.5_Rnor_6.0_rna.fna.gz. All of the datasets referenced in this study can be obtained upon reasonable request to the corresponding authors.

## Abbreviations

CMV: Controlled mechanical ventilation
VIDD: Ventilator-induced diaphragm dysfunction
miRNA: Microribonucleic acid
RNA-seq: RNA-sequencing
DE: Differentially expressed
GO: Gene oncology
KEGG: Kyoto encyclopedia of gene and genomes
BP: Biological process
CC: Cellular component
MF: Molecular function
RT-qPCR: Reverse transcription-quantitative PCR
