# Supplementary figures and images for: Altered expression of microRNAs in the rat diaphragm in a model of ventilator-induced diaphragm dysfunction after controlled mechanical ventilation
Source: BMC Genomics. 2021 Sep 18;22:671. doi: 10.1186/s12864-021-07970-y (PMC8449218; doi:10.1186/s12864-021-07970-y)

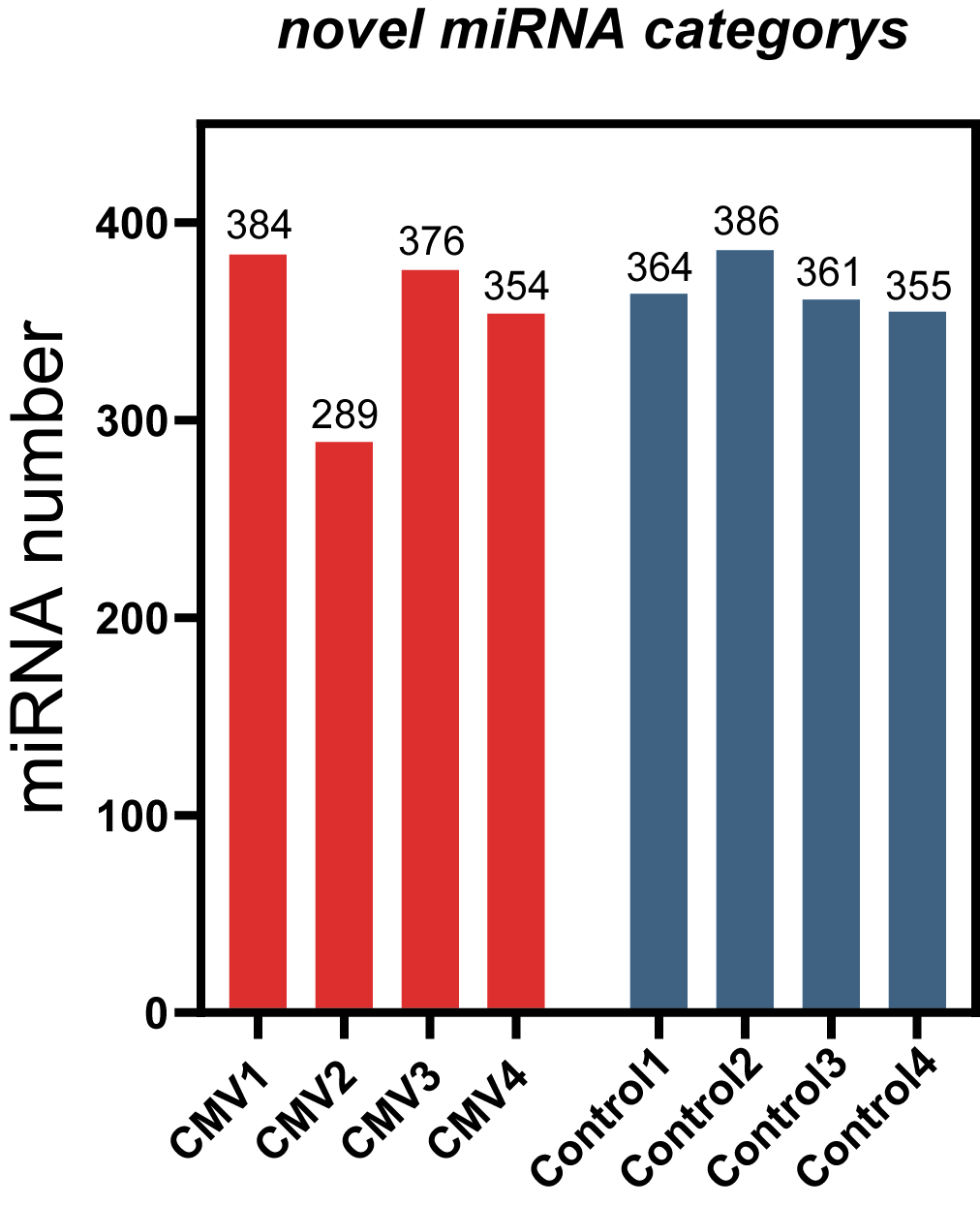

Supplement: Supplementary file 6 — Additional file 6: Figure S1. Novel miRNA categories. [file 12864_2021_7970_MOESM6_ESM.tif]

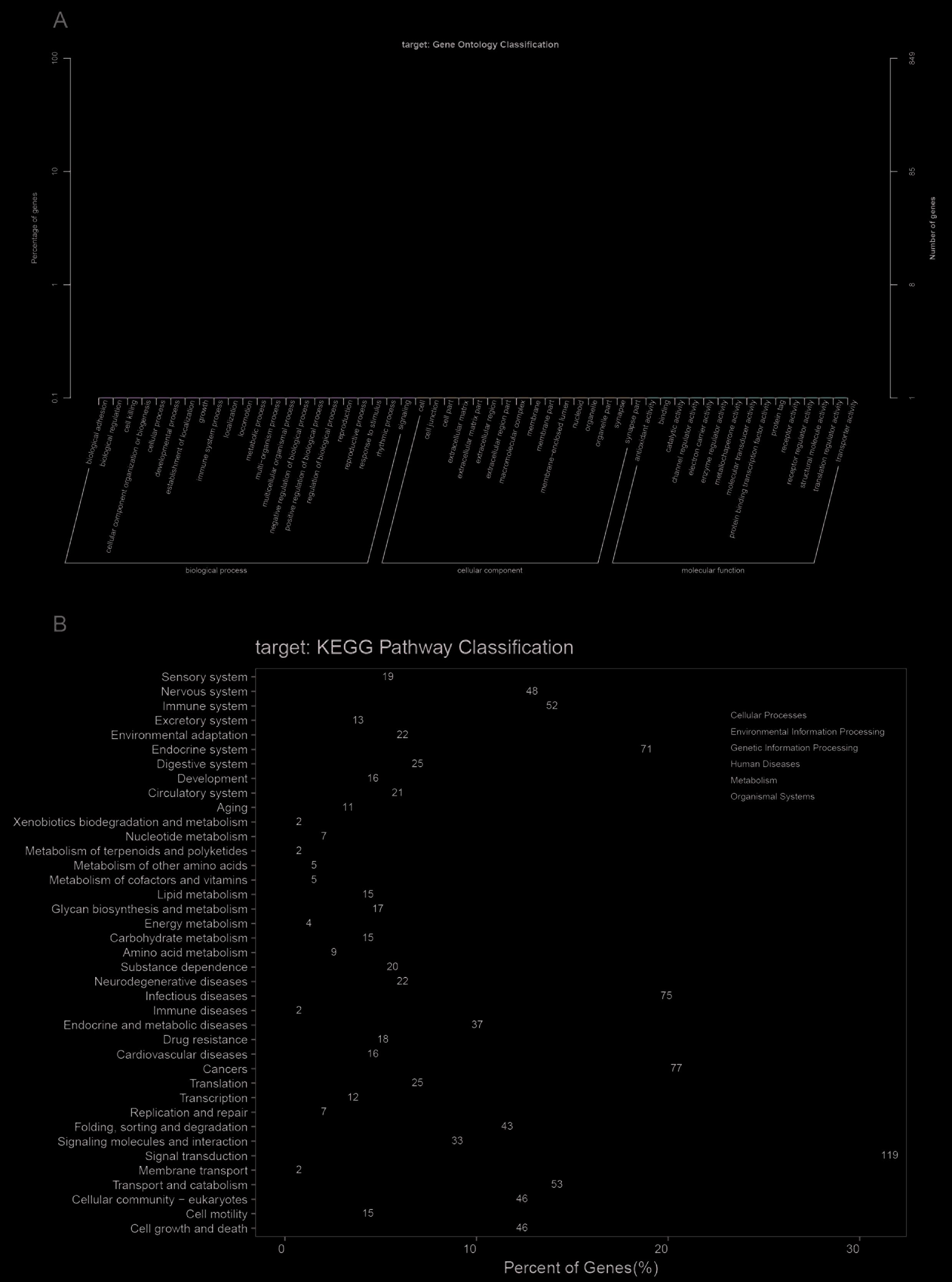

Supplement: Supplementary file 7 — Additional file 7: Figure S2. (A) GO level2 distribution of different miRNAs. (B) KEGG level2 distribution of different miRNAs. [file 12864_2021_7970_MOESM7_ESM.tif]
